# Genetic diversity and population structure in divergent German cattle selection lines on the basis of milk protein polymorphisms

**DOI:** 10.5194/aab-64-91-2021

**Published:** 2021-03-11

**Authors:** Lisa G. Hohmann, Christina Weimann, Carsten Scheper, Georg Erhardt, Sven König

**Affiliations:** Institute of Animal Breeding and Genetics, University of Giessen, 35390 Giessen, Germany

## Abstract

The aim of this study was to analyze the genetic structure of the casein cluster in eight selection lines of the Holstein Friesian (HF), German Simmental (GS) and German Black Pied cattle (“Deutsches Schwarzbuntes Niederungsrind”, DSN) breeds. A total of 2962 milk samples were typed at $\alpha_{s1}$-casein ($\alpha_{s1}$-CN), β-casein (β-CN), $\alpha_{s2}$-casein ($\alpha_{s2}$-CN) and κ-casein (κ-CN) loci using isoelectric focusing. The number of alleles per locus ranged from one ($\alpha_{s2}$-CN) to five (β-CN), and the average expected heterozygosity and polymorphic information content of all loci were 0.33 and 0.27, respectively. The unrooted dendrogram revealed that the selection lines of the endangered DSN breed were clearly separated from the HF and GS breeds due to their predominance of the β-CN A1 allele and the comprehensive haplotype BA1A (in the abbreviation of $\alpha_{s1}$-β-κ-CN). Temporal changes in allele distributions indicated decreasing genetic diversity at the casein loci, explaining the moderate level of genetic differentiation among selection lines (7.1 %). The variability of the casein should be exploited in future using breeding programs to select genetic lines for specific protein production in bovine milk but also to preserve biodiversity.

## 1 Introduction

Since domestication 8000–10 000 years ago, natural as well as man-made factors including geography, environment, culture and directional artificial selection contributed to cattle trait modifications phenotypically and genetically (Loftus et al., 1994). From a time perspective, in contrast to natural selection, artificial selection has the ability to change the genome rapidly. The consequence is a targeted displacement in allele frequencies, implying deviations from Hardy–Weinberg equilibrium (HWE) (Lachance, 2009). Two types of selection appear on the genomic level. Positive (Darwinian) selection promotes the spread of beneficial alleles, so that frequencies for these alleles increase and the selected alleles might be fixed over generations (Maynard Smith and Haigh, 1974; Kreitman, 2000). Negative or purifying selection hinders the spread of unfavorable alleles, causing decreasing allele frequencies up to the complete loss from the population (Kreitman, 2000). Selection not only affects the favored or unfavored mutations directly. In addition, selection causes a “hitchhiking” effect on the frequency of neutral alleles at linked loci (Maynard Smith and Haigh, 1974). The cattle genome therefore represents an opportunity for the identification of genetic variation that contributes to phenotypic diversity and for inferring genome responses to strong artificial selection. The different methods to detect selection signatures are based either on the distribution of allele frequencies, on the properties of haplotypes segregating within populations or on genetic differentiation between populations (reviewed by Hohenlohe et al., 2010).

Along with divergent selection criteria, the long-lasting intensive specific improvement of economically important traits contributed to the formation of diverse genetic lines within breeds. For example, artificial selection in the dual-purpose Simmental breed implied the establishment of divergent strains which are specialized for either milk or meat production (Campbell and Marshall, 2016). As the future requires promotion of more efficient sustainable livestock systems and utilization of greater proportions of non-human competitive products for animal feed, attention is given on adaptation to grazing systems (Delaby et al., 2018). Pasture-based systems reflect harsh environments, emphasizing the importance of animal traits associated with grazing behavior and robustness. Functional traits required under grazing include feed efficiency, health, fertility and longevity (Washburn and Mullen, 2014). In predestinated locations in Ireland or New Zealand, the development of grazing systems is accompanied by animal breeding and selection strategies on adaptation to local conditions (e.g., Lopez-Villalobos et al., 2000). The New Zealand total merit index favors robust, lightweight, long-living and efficient milk producing pasture converters (Jaeger, 2018). However, German Holstein (HF_G) cows have been selected for modern and large-scale indoor systems during decades, raising questions of possible genotype–environment interactions with impact on adaptation capabilities to harsh environments (König et al., 2005). In consequence, so-called “pasture breeding projects” were initiated in Germany (Brügemann et al., 2015; May et al., 2017), aiming at genetic line comparisons in grassland systems. Specific pasture-based selection lines within the HF_G breed were created by mating, e.g., HF_G cows with Holstein Friesian sires from New Zealand (HF_NZ). The close genetic relationships between selection lines with the same founder animals suggest genetic comparisons on the basis of milk protein compositions, in order to study effects of selection in dairy lines during the past decades.

Genes influencing milk yield and protein content are the casein genes *CSN1S1*, *CSN1S2*, *CNS2* and *CSN3*, encoding the proteins $\alpha_{s1}$-casein ($\alpha_{s1}$-CN), β-casein (β-CN), $\alpha_{s2}$-casein ($\alpha_{s2}$-CN) and κ-casein (κ-CN), respectively (Ng-Kwai-Hang et al., 1984). Several single-nucleotide polymorphisms (SNPs) of the casein genes change their protein sequences, implying different casein variants. A recent review of milk protein nomenclature (Gallinat et al., 2013) indicated 10 variants for $\alpha_{s1}$-CN (A, B, C, D, E, F, G, H, I, J), 15 for β-CN (A1, A2, A3, B, C, D, E, F, G, H${}^{1}$, H${}^{2}$, I, J, K, L), five for $\alpha_{s2}$-CN (A, B, C, D, E) and 14 for κ-CN (A, A1, B, B2, C, D, E, F${}^{1}$, F${}^{2}$, G${}^{1}$, G${}^{2}$, H, I, J) in *Bos genus*. The tight genetic linkage among the casein genes within a 250 kb cluster on chromosome 6 (BTA6) implies limited recombination and suggests the creation of casein haplotypes (Ferretti et al., 1990; Lien et al., 1993). Casein polymorphisms were used for the characterization of domesticated breeds and for tracing the evolutionary history (Caroli et al., 2009). Beja-Pereira et al. (2002) and Jann et al. (2004) provided evidence for a geographically associated distribution of casein haplotypes, and they identified a decline of genetic diversity for taurine breeds in Europe from the south to the north and from the east to the west. Mahé et al. (1999) discriminated between *Bos taurus* and *Bos indicus* origins at the milk protein level. Furthermore, casein genes harbor a number of variants with beneficial effects on milk production, milk composition and technological properties (reviewed by Caroli et al., 2009). Additionally, numerous studies (e.g., Ehrmann et al., 1997; Çardak et al., 2003) focused on the effects of polymorph milk proteins on the individual milk protein content. For example, Ng-Kwai-Hang et al. (1984) identified causal relationships between the homozygote genotypes BB of the respective casein $\alpha_{s1}$-CN and κ-CN with the protein and casein content of milk. Protein yield and protein percentage are included into the overall production index (RZM) for German dairy cattle since decades and have been used as a major selection criterion (König et al., 2007). In consequence, monitoring casein genetic variants is a useful tool to inferring signatures of selection.

To our knowledge, there are no studies addressing genetic diversity in individual selection lines – especially in pasture-based selection lines – based on alleles and haplotypes of the whole casein cluster. We hypothesize that divergent directions of positive selection (e.g., towards pasture ability, dairy or meat production) have altered allele and haplotype frequencies of the casein. Therefore, the aims of the present study were to (i) compare allele and haplotype frequencies across selection lines, (ii) study temporal changes in allele frequencies since the past 25 years and (iii) analyze genetic diversity between individual selection lines and evaluate effects of selection on casein frequencies.

## 2 Materials and methods

### 2.1 Animals

Milk samples from 2962 cows from first to third lactation of the Holstein Friesian (HF), German Black Pied cattle (“Deutsches Schwarzbuntes Niederungsrind”, DSN) and German Simmental (GS) breeds were collected in 2018. The samples were obtained from 50 small and medium-sized farms spread over Germany. Herd sizes ranged from 24 to 228 milking cows, with an average of 76 cows per farm.

**Table 1:** Description of selection lines of the Holstein Friesian (HF), German Black Pied (DSN) and German Simmental (GS) breeds and lactation production records for milk yield, fat percentage and protein percentage for the HF lines.

Selection line	Abbreviation	Description	Production	Number of
			records	cows
German Holstein (milk)	HF_G${}_{m}$	HF_G–HF_G sires with high breeding values for milk yield in pasture-based systems	9894 kg 3.99 % 3.31 %	64
German Holstein (pasture)	HF_G${}_{p}$	HF_G–HF_G sires selected for pasture conditions	8702 kg 4.21 % 3.45 %	50
Holstein Friesian (New Zealand)	HF_NZ	German Holstein cow (HF_G)–New Zealand sires	8003 kg 4.40 % 3.60 %	25
German Holstein (reference)	HF_G${}_{\mathrm{ref}}$	HF_G kept indoors	10 229 kg 4.04 % 3.36 %	1069
German Black Pied (east)	DSN${}_{\mathrm{east}}$	DSN from the new federal states of Germany		1158
German Black Pied (west)	DSN${}_{\mathrm{west}}$	DSN from the old federal states of Germany		293
German Simmental (milk)	GS${}_{m}$	GS; dual-purpose breed in milk production systems		124
German Simmental (beef)	GS${}_{b}$	GS; beef cattle breed with high value for meat production		179

The breeds were subdivided into eight selection lines based on divergent breeding strategies (Table 1). With regard to the HF breed, a total of four selection lines was considered. Three HF lines were established in the framework of the “German pasture genetics project” (Brügemann et al., 2015; May et al., 2017) considering a specific mating design in participating grazing herds. The first line in the grazing herds (HF_NZ) based on inseminations of HF_G cows with HF sires from New Zealand. The second line (HF_G${}_{p}$) was established considering mating between HF_G cows from the grazing herds with HF_G “pasture” sires. The selected HF_G pasture sires are suited to grazing conditions and represented favorable breeding values for traits that were important in New Zealand (i.e., small body size, high fat percentage, high non-return rate, short interval from calving to first insemination) (May et al., 2017). The third HF line (HF_G${}_{m}$) from the grazing herds included female offspring from mating of HF_G cows with HF_G sires representing outstanding breeding values for milk yield. The fourth HF line (HF_G${}_{\mathrm{ref}}$) considered HF_G cows from intensive indoor systems, i.e., herds with a strong selection focus on milk yield. Continuous selection strategies within lines contributed to production trait differences, especially for lactation milk yield and fat percentage as indicated in Table 1.

The local dual-purpose DSN cattle population is the founder breed of the modern HF population and has a long breeding history in the grassland region of East Frisia, Lower Saxony, Germany (Mügge et al., 1999). The DSN breeding goal considers both output traits milk and meat. DSN is defined as robust cattle under harsh environmental conditions and showed superiority over HF in terms of physiological traits (Al-Kanaan, 2016). Due to divergent breeding strategies under different housing conditions after World War II (separation into East and West Germany), two selection lines for DSN were considered (DSN${}_{\mathrm{east}}$ and DSN${}_{\mathrm{west}}$, respectively). For Simmental cattle, the most famous dual-purpose breed for milk and beef production in Germany, two selection lines were included: GS cows of the dual-purpose breed in milk production systems (GS${}_{m}$) and GS suckler cows as used in beef production systems (GS${}_{b}$).

### 2.2 Milk protein typing

Skimmed milk samples from 2962 cows were analyzed for milk protein polymorphisms of $\alpha_{s1}$-CN, $\alpha_{s2}$-CN, β-CN and κ-CN by isoelectric focusing in 0.3 mm thin polyacrylamide gels according to Seibert et al. (1985) and Erhardt (1989). This method describes the simultaneous separation of the known $\alpha_{s1}$-CN, β-CN, $\alpha_{s2}$-CN and κ-CN variants due to their isoelectric point and considers genetic variants which cannot be detected via commercial SNP chip applications.

### 2.3 Statistical analyses

Allele frequencies were calculated by direct counting, and HWE was tested by applying a $\chi^{2}$ test using the packages adegenet version 2.1.1 (Jombart, 2008; Jombart and Ahmed, 2011) and pegas (Paradis, 2010), as implemented in the software package R, version 2.14.2 (R Core Team, 2019). The polymorphic information content (PIC) was computed for each locus within and across populations using the R package polysat (Clark and Jasieniuk, 2011). The observed ($H_{o}$) and expected ($H_{e}$) heterozygosity were calculated using the R package adegenet. Wright's F-statistic parameters ($F_{\mathrm{IS}}$, $F_{\mathrm{IT}}$, $F_{\mathrm{ST}}$; Wright, 1965) describing the expected level of heterozygosity at various levels of population structure were calculated for each locus across all selection lines using the R package hierfstat (Goudet and Jombart, 2015). The most widely used fixation index ($F_{\mathrm{ST}}$) serves as a measure of population differentiation due to genetic structure. The overall inbreeding coefficient ($F_{\mathrm{IT}}$) measures the reduction in heterozygosity of an individual relative to the total population, whereas Wright's inbreeding coefficient ($F_{\mathrm{IS}}$) measures the reduction in heterozygosity of an individual due to non-random mating within its subpopulation (Wright, 1965). The R package hierfstat was also applied for the calculation of $F_{\mathrm{IS}}$ per population and loci. Negative $F_{\mathrm{IS}}$ values indicate heterozygote excesses and positive $F_{\mathrm{IS}}$ values imply a deficiency of heterozygotes, indicating a considerable level of inbreeding. Haplotypes were inferred using the software package PHASE version 2.1 (Stephens et al., 2001), in order to evaluate the haplotype variability within and among populations.

The standard genetic distance ($D_{s}$) according to Nei (1972) was calculated from haplotype frequencies using the R package adegenet. The unrooted dendrogram was constructed using the unweighted pair-group method with arithmetic mean (UPGMA) (Sneath and Sokal, 1973) to reconstruct phylogenetic relationships. The robustness of the phylogenies was evaluated by bootstrap values, considering 10 000 replications of resampling loci.

Discriminant analysis of principal components (DAPC) as implemented in the R packages ade4 (Bougeard and Dray, 2018) and adegenet was used to illustrate the admixture within the populations. In contrast to other common multivariate approaches (e.g., principal component analysis or factorial correspondence analysis), DAPC maximizes the separation between groups while minimizing variation within a group, providing a clear discrimination of pre-defined genetic groups (Jombart et al., 2010; Alves et al., 2015).

## 3 Results

### 3.1 Allele frequencies and test for Hardy–Weinberg equilibrium

A total of 11 alleles were detected in eight selection lines at four casein loci. The number of alleles per locus ranged from five (β-CN) over three (κ-CN) to two ($\alpha_{s1}$-CN) alleles. For $\alpha_{s2}$-CN, only the allele A was identified, so that the monomorphic locus $\alpha_{s2}$-CN was excluded from further analyses. Allele frequencies of the remaining casein loci $\alpha_{s1}$-CN, β-CN and κ-CN in the eight studied selection lines are presented in Table 2. For $\alpha_{s1}$-CN, all selection lines showed an average high frequency for the common B allele (97 %) and a minor allele frequency (MAF) of 3 % for the C allele. Only the selection lines HF_G${}_{p}$ (7 %), GS${}_{b}$ (6 %) and GS${}_{m}$ (6 %) showed a MAF larger than 3 % for the C allele. For β-CN, the variant A2 (53 %) was the predominant allele, but the two DSN subpopulations had a higher proportion of the A1 allele (in average 67.3 %). The highest frequency of A2 was found in HF_NZ (68 %). The A3 allele revealed highest frequencies in HF_G${}_{p}$ (7 %) and HF_NZ (2 %) but was zero in both GS subpopulations. The C allele of β-CN only occurred in GS${}_{b}$ (2 %) and GS${}_{m}$ (0.4 %) and thus represents a breed-specific allele for GS. With regard to κ-CN, the allele A had the largest frequency in most of the selection lines, but exceptions with a higher or equal frequency of the κ-CN B allele were HF_G${}_{p}$ and HF_NZ with 51 % and 50 %, respectively (Table 2). The highest frequency of the κ-CN E allele was found in HF_G${}_{\mathrm{ref}}$ (10 %). The rare allele C, which was detected by Erhardt (1993) in GS with a frequency of 0.02 %, was not identified in the sampled animals. In the $\chi^{2}$ test for HWE, three and five selection lines showed significant deviation (P < 0.05) for the β-CN and κ-CN loci, respectively, with corresponding degrees of freedom (d.f.) ranging from 1 to 6 (Table 2). The calculated $\chi^{2}$ value for $\alpha_{s1}$-CN was 0.57 on average (d.f. = 1), indicating HWE in all populations (P > 0.5, Table 2).

**Table 2:** Allele frequencies at the casein loci and tests for Hardy–Weinberg equilibrium with corresponding $\chi^{2}$ values from the $\chi^{2}$ test statistics in the studied selection lines. (Significant deviation from Hardy–Weinberg equilibrium at P<0.05 is indicated with *; n.s. indicates a non-significant deviation; d.f. indicates the degrees of freedom used in the $\chi^{2}$ test statistics).

	Selection lines${}^{1}$								Erhardt (1993)${}^{2}$		
	DSN${}_{\mathrm{west}}$	DSN${}_{\mathrm{east}}$	HF G${}_{\mathrm{ref}}$	HF G${}_{m}$	HF G${}_{p}$	HF NZ	GS${}_{b}$	GS${}_{m}$	Mean	HF_G	GS
$\alpha_{s1}$-CN											
B	0.987	0.993	0.987	0.988	0.932	0.978	0.945	0.944	0.969	0.989	0.887
C	0.013	0.007	0.013	0.012	0.068	0.022	0.055	0.056	0.031	0.011	0.113
$\chi^{2}$	0.03	0.03	0.23	0.00	0.28	0.01	3.5	0.44			
P	n.s.	n.s.	n.s.	n.s.	n.s.	n.s.	n.s.	n.s.			
d.f.	1	1	1	1	1	1	1	1			
β-CN											
A1	0.678	0.667	0.320	0.374	0.428	0.300	0.288	0.310	0.422	0.487	0.252
A2	0.291	0.311	0.640	0.624	0.481	0.679	0.611	0.646	0.534		0.657
A3	0.000	0.001	0.004	0.003	0.070	0.021	0.000	0.000	0.014	0.011	0.000
B	0.031	0.021	0.036	0.002	0.021	0.000	0.081	0.040	0.029	0.020	0.091
C	0.000	0.000	0.000	0.000	0.000	0.000	0.020	0.004	0.003	0.000	0.010
$\chi^{2}$	8.16	111.18	15.02	1.60	7.59	2.13	4.52	4.01			
P	*	*	*	n.s.	n.s.	n.s.	n.s.	n.s.			
d.f.	3	6	6	6	6	3	6	6			
κ-CN											
A	0.687	0.822	0.657	0.547	0.450	0.500	0.721	0.768	0.644	0.811	0.760
B	0.222	0.136	0.241	0.421	0.510	0.500	0.258	0.232	0.315	0.134	0.220
E	0.091	0.042	0.102	0.032	0.040	0.000	0.021	0.000	0.041	0.055	0.000
C	0.000	0.000	0.000	0.000	0.000	0.000	0.000	0.000	0.000	0.000	0.020
$\chi^{2}$	34.44	14.29	7.30	12.48	12.68	9.01	1.11	1.67			
P	*	*	n.s.	*	*	*	n.s.	n.s.			
d.f.	3	3	3	3	3	1	3	1			

${}^{1}$ Abbreviations for selection lines are given in Table 1. ${}^{2}$ For comparisons of present results with historic data, allele frequencies of the HF_G and GS breeds from the study by Erhardt (1993) were considered.

### 3.2 Genetic variation of casein loci

The breed- and casein-wise estimates of $H_{o}$, $H_{e}$ and $F_{\mathrm{IS}}$ as well as the PIC are presented in Table 3. Across selection lines, the locus β-CN had the highest $H_{e}$ (0.53) and the highest PIC (0.42), while the locus $\alpha_{s1}$-CN displayed the lowest $H_{e}$ and PIC values (both 0.03). The κ-CN locus was characterized by intermediate $H_{e}$ (0.42) and PIC (0.37) values. Mean $H_{o}$ values for each locus were 0.03 for $\alpha_{s1}$-CN, 0.46 for β-CN and 0.41 for κ-CN. Within selection lines, the level of genetic variation was highest for HF_G${}_{p}$ (average $H_{e}$ overall loci = 0.41) and lowest for DSN${}_{\mathrm{east}}$ (average $H_{e}$ overall loci = 0.26). The $F_{\mathrm{IS}}$ values ranged from -0.07 (HF_G${}_{p})$ to 0.14 (GS${}_{b}$) at $\alpha_{s1}$-CN, from -0.23 (HF_NZ) to 0.27 (DSN${}_{\mathrm{east}}$) at β-CN and from -0.74 (HF_NZ) to 0.14 (DSN${}_{\mathrm{west}}$) at κ-CN. The negative $F_{\mathrm{IS}}$ values of some breeds indicated an excess of heterozygotes.

**Table 3:** The breed- and casein-wise estimates of observed ($H_{o}$) and expected heterozygosity ($H_{e}$), Wright's inbreeding coefficient ($F_{\mathrm{IS}}$; Wright, 1965) and polymorphic information content (PIC).

	$\alpha_{s1}$-CN				β-CN				κ-CN			
Selection lines	$H_{o}$	$H_{e}$	$F_{\mathrm{IS}}$	PIC	$H_{o}$	$H_{e}$	$F_{\mathrm{IS}}$	PIC	$H_{o}$	$H_{e}$	$F_{\mathrm{IS}}$	PIC
DSN${}_{\mathrm{west}}$	0.02	0.02	-0.01	0.020	0.53	0.45	-0.17	0.374	0.41	0.47	0.14	0.413
DSN${}_{\mathrm{east}}$	0.01	0.01	-0.01	0.010	0.34	0.46	0.27	0.368	0.30	0.30	0.02	0.278
HF_G${}_{m}$	0.02	0.02	0.00	0.020	0.45	0.48	0.07	0.373	0.76	0.51	-0.44	0.413
HF_G${}_{p}$	0.14	0.13	-0.07	0.122	0.60	0.58	-0.03	0.490	0.78	0.52	-0.48	0.429
HF_NZ	0.04	0.04	0.00	0.038	0.56	0.45	-0.23	0.364	0.88	0.50	-0.74	0.375
HF_G${}_{\mathrm{ref}}$	0.03	0.03	-0.01	0.020	0.53	0.49	-0.09	0.401	0.50	0.50	-0.01	0.437
GS${}_{b}$	0.10	0.11	0.14	0.106	0.48	0.53	0.10	0.468	0.54	0.41	-0.05	0.291
GS${}_{m}$	0.11	0.11	-0.06	0.106	0.52	0.49	-0.08	0.398	0.40	0.35	-0.11	0.370
Total	0.03	0.03	0.001	0.03	0.46	0.53	-0.02	0.42	0.41	0.42	-0.23	0.37

The fixation coefficients of subpopulations within the total population, measured as $F_{\mathrm{ST}}$ value for the three loci $\alpha_{s1}$-CN, β-CN and κ-CN, varied from 0.016 ($\alpha_{s1}$-CN) to 0.080 (β-CN), with a mean of 0.071. It means that 7.1 % of the total genetic variation in the selection lines corresponds to genetic differences among populations, while the remaining 92.9 % explained differences among individuals within population. Additionally, results of F statistics revealed on average an excess of heterozygotes of 11.3 % for each of the analyzed subpopulations ($F_{\mathrm{IS}})$ and 3.4 % in the whole population ($F_{\mathrm{IT}}$). In comparison to the negative $F_{\mathrm{IS}}$ values of κ-CN (-0.233) and β-CN (-0.017) among the eight selection lines, the casein locus $\alpha_{s1}$-CN showed a deficit of heterozygotes due to its positive $F_{\mathrm{IS}}$ value (0.001).

### 3.3 Casein haplotype distributions

Table 4 represents the results of the haplotype analysis of the $\alpha_{s1}$–β–κ-CN cluster (in order according to their location on BTA6). A total of 13 haplotypes was identified. More than 80 % of all individuals carried one of the BA1A, BA2A or BA2B haplotypes (abbreviation of the specific combination of $\alpha_{s1}$–β–κ-CN alleles), with mean frequencies of 35 %, 32 % or 20 %, respectively. DSN${}_{\mathrm{east}}$, DSN${}_{\mathrm{west}}$ and HF_G${}_{p}$ revealed the highest frequencies for BA1A, while the most frequent haplotype for the remaining selection lines was BA2A (Table 4).

**Table 4:** Haplotype frequencies in the studied selection lines.

Haplotype${}^{1}$	DSN${}_{\mathrm{west}}$	DSN${}_{\mathrm{east}}$	HF_G${}_{\mathrm{ref}}$	HF_G${}_{m}$	HF_NZ	HF_G${}_{p}$	GS${}_{b}$	GS${}_{m}$	All${}^{2}$
BA1A	0.50	0.57	0.21	0.34	0.28	0.33	0.27	0.31	0.35
BA1B	0.11	0.07	0.03	0.02	0.02	0.08	0.01	0.004	0.04
BA2A	0.18	0.26	0.44	0.35	0.38	0.19	0.34	0.38	0.32
BA2B	0.09	0.05	0.18	0.25	0.30	0.29	0.20	0.21	0.20
BA1E	0.08	0.04	0.09	0.01	0.00	0.02	0.01	0.00	0.03
BBA	0.01	0.001	0.001	0.00	0.00	0.00	0.06	0.03	0.01
CA2A	0.01	0.001	0.01	0.00	0.00	0.00	0.06	0.06	0.02
BBB	0.01	0.02	0.04	0.01	0.00	0.02	0.01	0.01	0.02
BA2E	0.01	0.00	0.01	0.02	0.00	0.00	0.01	0.00	0.01
CA3A	0.00	0.001	0.004	0.01	0.02	0.07	0.00	0.00	0.01
BCB	0.00	0.00	0.00	0.00	0.00	0.00	0.02	0.004	0.003
CA2B	0.00	0.003	0.002	0.00	0.00	0.00	0.003	0.00	0.001
BBE	0.002	0.00	0.00	0.00	0.00	0.00	0.003	0.00	0.001

${}^{1}$ In the abbreviation of $\alpha_{s1}$–β–κ-CN. ${}^{2}$ Average haplotype frequencies across all selection lines.

### 3.4 Genetic distances and population structure

**Figure 1:**
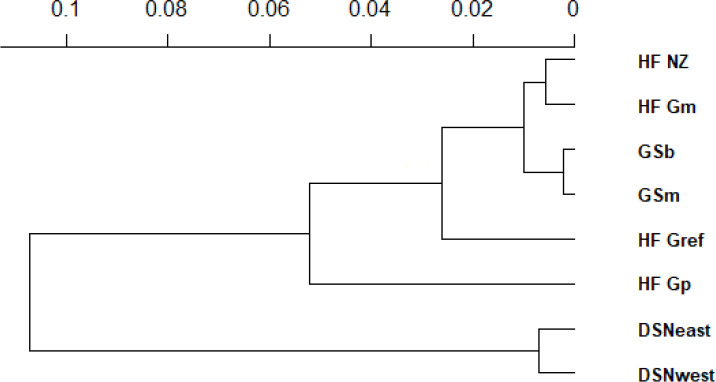
Unrooted dendrogram with bootstrap support using Nei's standard genetic distances ($D_{S})$ (Nei, 1972). The x axis represents the genetic distances between the eight studied selection lines.

**Figure 2:**
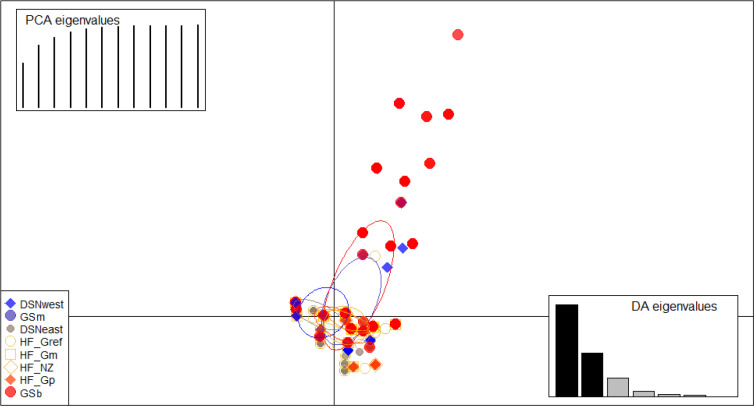
Scatterplot of the discriminant analysis of principal components (DAPC) based on casein haplotype frequencies. Eight selection lines are plotted according to the eigenvectors corresponding to the first (56 %) and second (27 %) linear discriminants. Each circle represents a cluster and each dot represents an individual.

The matrix of Nei's $D_{s}$ among the studied selection lines is presented in Table 5. We identified a very close relationship between GS${}_{b}$ and GS${}_{m}$ (0.004). A close relationship was also found between HF_G${}_{m}$ and HF_NZ (0.011), followed by HF_G${}_{m}$ and GS${}_{m}$ (0.013). The selection line HF_G${}_{\mathrm{ref}}$ was the most divergent from the two DSN subpopulations: 0.318 in relation to DSN${}_{\mathrm{west}}$ and 0.290 in relation to DSN${}_{\mathrm{east}}$. The pasture-based selection lines HF_G${}_{m}$, HF_G${}_{p}$ and HF_NZ showed a close relationship, as documented by low values for $D_{s}$ ranging from 0.011 to 0.068. The HF_G${}_{\mathrm{ref}}$ subpopulation distanced itself from the pasture based subpopulations with $D_{s}$ values up to 0.193. In the UPGMA dendrogram (Fig. 1), two main clusters and two subclusters were identified. In the first main cluster, HF_NZ, HF_G${}_{m}$, GS${}_{b}$, GS${}_{m}$ and HF_G${}_{\mathrm{ref}}$ were placed together, while HF_G${}_{p}$ was allocated to the other subcluster. Both DSN subpopulations (DSN${}_{\mathrm{east}}$, DSN${}_{\mathrm{west}})$ were placed in the second main cluster. Finally, the DAPC was used to show the genetic admixture between the selection lines. The respective results are shown in Fig. 2. For the best discrimination of haplotypes into pre-defined clusters, DAPC was run using 12 principal components and seven discriminant functions. The first two linear discriminants, which are illustrated in the scatterplot, contributed to 56 % and 27 % of the total variation, respectively. The first linear discriminant separated DSN and HF populations, whereas the second linear discriminant distinguished between the Simmental subpopulations from all other selection lines.

**Table 5:** Matrix of Nei's standard genetic distance ($D_{s})$ (Nei, 1972) obtained from the haplotype frequencies.

	DSN${}_{\mathrm{west}}$	HF_G${}_{m}$	HF_NZ	HF_G${}_{p}$	GS${}_{b}$	GS${}_{m}$	DSN${}_{\mathrm{east}}$	HF_G${}_{\mathrm{ref}}$
DSN${}_{\mathrm{west}}$	0.000	0.167	0.268	0.155	0.210	0.203	0.014	0.318
HF_G${}_{m}$		0.000	0.011	0.056	0.019	0.013	0.152	0.061
HF_NZ			0.000	0.068	0.026	0.022	0.253	0.053
HF_G${}_{p}$				0.000	0.102	0.104	0.188	0.193
GS${}_{b}$					0.000	0.004	0.190	0.050
GS${}_{m}$						0.000	0.174	0.044
DSN${}_{\mathrm{east}}$							0.000	0.290
HF_G${}_{\mathrm{ref}}$								0.000

## 4 Discussion

### 4.1 Temporal changes of milk protein polymorphisms

Temporal changes in allele frequencies of milk protein polymorphisms in the HF and GS common cattle breeds are evident when comparing results from the present study with allele frequencies for the same breeds 25 years ago (Erhardt, 1993; Table 2). The 25-year period reflects six generations of mating and selection schemes, with an additional possible impact of random genetic drift. At the β-CN locus, frequencies for the A2 allele were larger in the present than in the historical data, in particular for HF cows. In this regard, Erhardt (1993) detected an A2 allele frequency of 49 % in HF_G, but in the present study, the average allele frequency across the HF populations was 60 %. Chessa et al. (2019) observed a similar trend in temporal changes for A2 in Italian Holstein dairy cattle. Frequencies for A2 were 38.9 %, 49.0 %, 53.1 % and 55.7 % for cows born in 1990, 2000, 2010 and after 2010, respectively. Freyer et al. (1999) and Bech and Kristiansen (1990) reported a favorable impact of the A2 allele on milk and protein yield. Hence, ongoing selection of bulls and cows according to genetic merits for milk or protein yield indirectly increased the A2 variant for β-CN. The relatively high A2 frequencies in the “milk lines” HF_G${}_{\mathrm{ref}}$ (64 %) and GS${}_{m}$ (65 %) support such hypothesis. Another explanation addresses the relation of A1 milk consumption with the release of the opioid peptide β-casomorphin-7, which may play a role in the development of some human diseases (i.e., ischemic heart disease, type 1 diabetes) (Tailford et al., 2003; Kamiński et al., 2007; Cieślińska et al., 2012; Sheng et al., 2019). As the production of milk with special nutrition properties (i.e., hypoallergenic milk) benefits from the A2 variant of bovine β-CN (De Noni, 2008), farmers are encouraged to select favorable alleles for milk production in niche markets. Additionally, A2 variant information for HF_G sires recently is given in sire catalogues, public journals and discussion forums (Gödert et al., 2017). Nevertheless, the pasture-based genetic line HF_NZ revealed the highest frequency with 68 % for the A2 allele. On the one hand, this may be the result of crossing with the breed Jersey, which generally displays a high frequency of 67 % for β-CN A2 (Erhardt, 1993). In New Zealand, the crosses between HF and Jersey generated the so-called “kiwi cross”, a new synthetic breed with favorable values for milk composition traits (Rowarth, 2013; Buckley et al., 2014; Mogollón-García et al., 2020). Another explanation might be the intensive selection for the A2 variant as initiated by the “a2 Milk Company” founded in New Zealand (The a2 Milk Company, 2020). The “a2 Milk Company” initiated a milk marketing program, considering only cows carrying the homozygous β-CN genotype A2A2. Up to now, there have been no progresses regarding active marketing strategies for bovine milk with defined milk protein variants (e.g., A2 milk) in Germany (Gödert et al., 2017).

In contrast to the increasing frequencies of the A2 allele, the β-CN A1 allele declined with progressing time in all selection lines, apart from HF_G${}_{p}$. The genetic line HF_G${}_{p}$ reflects the A1 and A2 allele frequencies as identified by Erhardt (1993) in HF_G cows. Such a result indicates genomic characteristic similarities of low yielding HF_G cows from low input systems with the broad HF population 25 years ago.

With regard to κ-CN, frequencies of the B allele increased in all populations with progressing time. The B allele frequency in HF_G was 13 % in 1993 (Erhardt, 1993) but increased to 39 % (average from all HF selection lines). The increasing frequency may be due to the positive effect of the κ-CN B allele on milk protein percentage and therefore its favorable cheese-making properties (Hallén et al., 2008; Heck et al., 2009; Mohammadi et al., 2013). Such association stimulated interest in using casein polymorphism in marker-assisted selection schemes to improve milk performance traits in farm animals (Kumar et al., 2006). Simultaneously, the rare allele C was suppressed until its complete loss from the subpopulations. Departures from HWE in both loci (β-CN and κ-CN) reflect temporal changes, i.e., increasing frequencies of the favorable alleles A2 (β-C) and B (κ-CN), due to the impact of selection (Lachance, 2009).

For $\alpha_{s1}$-CN, frequencies in HF did not differ between present and historic data, because high frequencies of the $\alpha_{s1}$-CN B allele, close to fixation, were already reported by Erhardt (1993). In GS, the B allele frequency increased from 89 % (Erhardt, 1993) towards fixation (94 %; Table 2).

With regard to the $\alpha_{s2}$-CN locus, only the allele A was identified in all selection lines. The D allele is rather common in French breeds (e.g., Montbéliarde) (Grosclaude et al., 1979) but was also described for HF and GS with low frequencies of 0.2 % and 2 %, respectively (Erhardt, 1993; Meier et al., 2019). Such a loss of rare alleles (e.g., $\alpha_{s2}$-CN D allele, κ-CN C allele) indicates genetic drift, a mechanism of evolution in which allele frequencies change over generations by chance (Hartl and Clark, 2007), with an impact on decreasing genetic diversity.

### 4.2 Genetic diversity parameters

Among selection lines, HF_G${}_{p}$ displayed the highest gene diversity over all loci (average $H_{e}$ overall loci = 0.41). Alternative selection of HF in grazing systems with a focus on a broad pattern of functional traits including especially female fertility and somatic cells might explain their variability at protein loci. Additionally, observed (but rather limited) genetic exchange with DSN contributed to genetic diversity. The $H_{e}$ for each locus in the reference line HF_G${}_{\mathrm{ref}}$ is in agreement with the commercial Portuguese HF population (Beja-Pereira et al., 2002). The lowest values for $H_{e}$ across all loci were observed for DSN${}_{\mathrm{east}}$, which might be due to the larger inbreeding increase in the DSN east subpopulation compared to the subset for DSN cows from former West Germany (Jaeger et al., 2018a). As the deficiency of heterozygotes is an indication of inbreeding, the positive $F_{\mathrm{IS}}$ values for DSN${}_{\mathrm{east}}$ at both loci (β-CN and κ-CN) (0.27 and 0.02, respectively) underline this assumption. An explanation for the mating of closely related animals in the past DSN${}_{\mathrm{east}}$ is the restricted gene flow from foreign countries in the former German Democratic Republic. In contrast, in DSN${}_{\mathrm{west}}$, sires from the Netherlands have been used in the period from 1970 to 1980 (Jaeger et al., 2018a). Nevertheless, also for DSN${}_{\mathrm{west}}$, the diversity measurement ($H_{e}=0.31$) suggests a general small effective population size for DSN, reflecting a small real population with only 2800 registered cows in Germany (Rinderproduktion Berlin-Brandenburg GmbH, 2016). A decreasing population size is a major cause for losses in genetic diversity (Kantanen et al., 1999). In such context, Jaeger et al. (2018b) calculated an increase of inbreeding per year in DSN of 0.1 %, implying a rather small effective population size of 85 animals.

### 4.3 Relationships between selection lines

In the UPGMA dendrogram, the genetically closely related subpopulations of the DSN breed (DSN${}_{\mathrm{east}}$, DSN${}_{\mathrm{west}}$; $D_{S}=0.014$) built their own cluster, clearly differentiated from the remaining selection lines. The majority of selection lines (i.e., selection lines within HF and GS) revealed the highest frequency for the haplotype BA2A, which was also detected for Italian Friesian cattle (Boettcher et al., 2004). In contrast, in both DSN subpopulations, BA1A was the most frequent casein haplotype with a frequency up to 57 %. The predominant impact of such chromosomal segment and especially of the A1 allele (67.3 % on average for both DSN subpopulations) is in agreement with results of Ng-Kwai-Hang et al. (1984) and Meier et al. (2019). Generally, in addition to DSN, breeds originating from northern Europe, including European Red cattle (Bech and Kristiansen, 1990), Black and White Lowland breeds (McLean et al., 1984) and Danish Red cattle (Meier et al., 2019), showed highest frequencies for the A1 allele and the corresponding haplotypes. These results indicate that the β-CN A1 allele is a major characteristic for breeds with a certain geographic location in Nordic countries. A further explanation for genomic similarities in Nordic breeds including DSN addresses identical breeding objectives towards a dual-purpose phenotype. A shared characteristic of Nordic breeds and DSN is the similarity in fat and protein percentages (Meier et al., 2019).

The average genetic distance of 0.30 between the founder DSN breed (DSN${}_{\mathrm{east}}$, DSN${}_{\mathrm{west}})$ and the modern HF_G${}_{\mathrm{ref}}$ population indicates the breeding particularities in both lines in the past decades. The robust DSN cattle were subject of extensive breeding mostly in pasture-based production systems, predominantly considering mating with natural service sires. In contrast, HF_G${}_{\mathrm{ref}}$ cows were intensively selected for milk yield, indirectly favoring casein haplotypes as already reported in goats (Grosclaude and Martin, 1997). The results for Nei's $D_{S}$ indicate a close relationship between both DSN subpopulations and HF_G${}_{p}$. The predominance of the A1 allele in HF_G${}_{p}$ makes them more similar to DSN${}_{\mathrm{east}}$ and DSN${}_{\mathrm{west}}$ than to the current HF_G${}_{\mathrm{ref}}$ population. This might be due to the higher genetic percentage of DSN in their ancestors, as the genetic pasture line was selected for robust animals (Jaeger et al., 2018a, b). The high frequencies for the $\alpha_{s1}$-CN C allele in HF_G${}_{p}$ (7 %) and both DSN subpopulations (6 %) support such hypothesis.

Genetic distances were observed between HF selection lines, as they clustered separately. With regard to allele frequencies at the κ-CN locus, some specific patterns in selection lines were noticed. First, the selection lines HF_G${}_{p}$, HF_NZ and HF_G${}_{m}$ from the grazing herds displayed the highest allele frequencies for the favorable κ-CN B allele (51 %, 50 % and 42 %, respectively). The importance of specific breeding goal traits differed in divergent feeding systems (Washburn and Mullen, 2014; Delaby et al., 2018). In pasture-based systems, the focus of selection has emphasized fertility, fitness and robustness. The prevalence of the B allele in pasture-based selection lines may be the result of indirect selection for these traits. In this regard, Hiendleder et al. (2003) detected quantitative trait locus (QTL) linked with the milk protein genes on BTA6 for udder quality and limb conformation (e.g., quality of feed and leg), being traits reflecting the pasture ability.

In both GS subpopulations (GS${}_{b}$, GS${}_{m}$), we identified a private allele in the β-CN C variant and therefore the breed-specific haplotype BCB. This is in agreement with results by Çardak (2005), who found the C allele with a frequency of 2.3 % in Simmental cows but not in HF. The occurrence of β-CN C explains the lowest $D_{A}$ between GS${}_{b}$ and GS${}_{s}$ as well as their differentiation from the other selection lines (Fig. 1). The breed-specific C allele may be linked to a favorable mutation on BTA6 for carcass and body weight, promoting the breeding value for beef production in a dual-purpose breed like GS. In this regard, QTL for growth traits (i.e., body length, carcass weight) have been detected within the *NCAPG* gene located on BTA6 in local beef cattle breeds (e.g., Chinese Qingchuan and Japanese Black and Brown beef cattle), indicating overlapping mechanisms of bone and muscle growth with lipid deposition (Setoguchi et al., 2009; Liu et al., 2015). Furthermore, the gene *SPP1* on BTA6 was associated with body weight in Polish Holstein Friesian cattle (Pareek et al., 2008). In a functional genomic approach, Sheehy et al. (2009) suggested *SPP1* as an important regulator of bovine milk protein gene expressions, explaining the possible link between the casein and *SPP1*.

### 4.4 Genetic differentiation among selection lines

In the present study, the average $F_{\mathrm{ST}}$ among selection lines was 7.1 %, reflecting a moderate level of population differentiation (Hartl and Clark, 2007). Hence, 7 % of the total genetic variation corresponds to selection line particularities, and the remaining 93 % is due to individual differences. The illustration for DAPC (Fig. 2) indicates that the selection lines do not clearly distinguish divergent clusters. The DAPC visualizes a high admixture between the subpopulations. We only identified a separation between the DSN and HF subpopulations along the linear discriminant 1 and a separation between the Simmental subpopulations with the remaining selection lines. The slight genetic variation among subpopulations might be a result of the decreased variability at the casein loci, which is indicated by the average PIC of 0.27 over all loci. Genotyping of the casein genes (e.g., β-CN and κ-CN) is of increasing relevance for practical breeding and selection, also from a genetic diversity monitoring perspective.

## 5 Conclusions

The results of the present study indicate that different selection strategies (e.g., pasture ability, meat or dairy production) indirectly contributed to the variability of the casein polymorphisms linked to milk production traits. The selection lines of the endangered DSN breed showed the lowest gene diversity and clearly separated from the HF and GS breeds due to their predominance of the β-CN A1 allele. The pasture-based selection lines of the HF breed carried the favorable κ-CN B allele with highest frequency, which is related to a higher protein content in milk. Temporal changes in allele distributions reflect that casein loci or selected mutations in close proximity to the casein underlie selective breeding. Fixation of alleles and results for evaluated indicators of heterozygosity (e.g., $H_{e}$, $F_{\mathrm{ST}}$, $F_{\mathrm{IS}}$) showed diversity loss at the casein loci. The present study revealed differences in allele frequencies at casein loci across selection lines, indicating breeding potential for specific milk markets. Furthermore, genetic milk protein variants can be used to monitor genetic diversity.

## Data Availability

The data that support the findings of this study are available from the authors upon reasonable request.
